# Vincristine as an Adjunct to Therapeutic Plasma Exchange for Thrombotic Thrombocytopenic Purpura: A Single-Institution Experience

**DOI:** 10.4274/balkanmedj.2017.1215

**Published:** 2018-11-15

**Authors:** Seniz Öngören, Ayşe Salihoğlu, Tuğçe Apaydın, Sevil Sadri, Ahmet Emre Eşkazan, Muhlis Cem Ar, Tuğrul Elverdi, Zafer Başlar, Yıldız Aydın, Teoman Soysal

**Affiliations:** 1 Department of Internal Medicine, Division of Hematology, İstanbul University Cerrahpaşa School of Medicine, İstanbul, Turkey; 2 Department of Internal Medicine, İstanbul University Cerrahpaşa School of Medicine, İstanbul, Turkey

**Keywords:** Plasma exchange, purpura, thrombotic thrombocytopenic, vincristine

## Abstract

**Background:**

Thrombotic thrombocytopenic purpura is a potentially life-threatening condition. Although the introduction of therapeutic plasma exchange has reduced mortality rates from over 90% to 10%-20%, approximately 40% of patients relapse, and outcomes may be fatal in refractory patients. There is clearly a need for additional therapeutic approaches.

**Aims:**

To describe the outcomes of relapsed/refractory thrombotic thrombocytopenic purpura patients treated with vincristine as an adjunct to therapeutic plasma exchange.

**Study Design:**

Cross-sectional study.

**Methods:**

The medical records of all relapsed/refractory patients with thrombotic thrombocytopenic purpura treated with vincristine adjunct to therapeutic plasma exchange between October 2000 and December 2016 were retrospectively reviewed. Diagnosis of thrombotic thrombocytopenic purpura was based on clinical history, physical examination, and laboratory examinations. Patient demographics, laboratory findings, initial date and duration of therapeutic plasma exchange, dosage and time of administration of vincristine, and outcomes were recorded.

**Results:**

The study included 15 patients [median age: 37 years (range: 26-65); 7 women and 8 men] with either relapsed or refractory thrombotic thrombocytopenic purpura who were treated with vincristine as an adjunct to therapeutic plasma exchange for a total of 22 episodes. Eighty-seven percent of patients achieved remissions in 20 of 22 episodes, with a median duration of remission of 29.5 months (range: 3-105). After a median follow-up of 55 months, 11 patients were alive. Vincristine was well tolerated with no safety concerns.

**Conclusion:**

Vincristine offers a reasonable option for the treatment of patients with relapsed/refractory thrombotic thrombocytopenic purpura. Further studies evaluating vincristine in the front-line setting and in the relapsed/refractory setting are needed to validate the role of vincristine in thrombotic thrombocytopenic purpura patients.

Thrombotic thrombocytopenic purpura (TTP) is characterized by microangiopathic hemolytic anemia, thrombocytopenia, and organ dysfunction. ADAMTS13 (a disintegrin and metalloproteinase with a thrombospondin type 1 motif, member 13) plays a key role in the pathogenesis of TTP. This metalloproteinase cleaves the ultra-large von Willebrand factor (vWF) multimers, and its deficiency results in accumulation of the multimers in the circulation, leading to formation of platelet thrombi. As a result, multiorgan ischemia and dysfunction occur, with life-threatening complications. Antibodies of the immunoglobulin G type against ADAMTS13 have been shown to be the underlying pathogenetic mechanism in the majority of cases (1). Therapeutic plasma exchange (TPE) is the primary treatment for patients with TTP. Its mechanism of action includes replacement of the missing ADAMTS13 and removal of autoantibodies. A challenge with corticosteroids might help by inhibiting autoantibody production by B cells, especially for patients who fail to respond to TPE initially. Although rates of mortality from TTP have decreased after the introduction of TPE over the last 3 decades, TTP still has an unacceptably high mortality rate of 10%-20%. There is clearly a need for additional therapeutic approaches. In the front-line setting, the anti-CD20 monoclonal antibody rituximab has been reported to result in shorter hospitalizations and fewer relapses (2). Alternative immunosuppressive therapies include vincristine, cyclosporine, mycophenolate mofetil, and cyclophosphamide (3). Vincristine is an antitumor vinca alkaloid agent indicated in the treatment of a variety of malignancies. It interacts with the microtubular and spindle contractile proteins in the S phase of the cell cycle, causing mitotic arrest and cell death. Suggested mechanisms of action also include alteration of glycoprotein receptor expression on the platelet surface, resulting in decreased attachment of platelets to vWF multimers and reduction in platelet aggregation. Furthermore, immunomodulation at the endothelial cell level has also been reported. It is the only antimitotic immunosuppressant agent with no major toxic effects on the marrow megakaryopoiesis (4). Side effects include sensory neuropathy, muscle weakness, paralytic ileus, leukopenia, and transient alopecia (1). Vincristine has been used in relapsed or refractory TTP, and many studies support a potential role for vincristine in the treatment of TTP (5,6,7,8,9,10,11,12,13,14,15,16,17,18).

This study includes a retrospective evaluation of vincristine use in patients with TTP treated in a tertiary referral center over a 16-year period with the aim of reviewing the efficacy and tolerability of vincristine.

## MATERIALS AND METHODS

A retrospective analysis of medical records of all TTP patients treated at our center between October 2000 and December 2016 was conducted. The patients who received vincristine were further evaluated. Patient demographics including age and sex, medical history, presenting signs and symptoms, and laboratory values [complete blood count, peripheral blood smear examination for schistocytes, reticulocyte count, lactate dehydrogenase (LDH), haptoglobin, total and indirect bilirubin, coagulation tests, Coombs tests, creatinine and ADAMTS13 antigen, activity and antibody levels, if performed] were derived from the medical records. Diagnosis of TTP was based on clinical history, physical examination, and laboratory examinations. The initial date and duration of TPE, the dosage and time of administration of vincristine, and outcomes were recorded. Ethical approval for the study was obtained from the local ethics committee (Ethical committee approval number: 3984/2018). Informed written consent was obtained from all patients. TPE with 1 plasma volume was initiated using standard fresh frozen plasma and continued until a platelet count of >150.000/mm^3^ was achieved for 2 consecutive days. TPE was then discontinued without tapering unless exacerbation occurred. In the case of exacerbation, daily TPE was resumed, and tapering was considered when a response occurred and stopping TPE was appropriate.

### Administration of the drug

Vincristine was administered intravenously 1 or 2 mg on day 1, followed by 1 mg on days 4 and 7 following TPE. A second course was given after a 1-week interval.

### Definitions of clinical outcomes (19)

The date of diagnosis was designated as the date of the first TPE. Time to response was defined as the time to recovery of a platelet count ≥150.000/mm^3^ for 2 consecutive days. Refractory disease was defined as a lack of platelet recovery after 4-7 days of TPE. Disease exacerbation was defined as relapsing thrombocytopenia following a response and requiring reinitiation of daily TPE after ≥1 day but ≤30 days of no TPE treatment. Disease exacerbation can also occur while on TPE. Remission was defined as the absence of a need for TPE for ≥30 days. Relapse was the recurrence of TTP following a period of remission. Time to relapse was defined as the time from the discontinuation of the last treatment to relapse of TTP. Death occurring within 30 days of completion of TPE was defined as TTP-associated death. The duration of follow-up was calculated from the time of the first vincristine administration to the last clinical follow-up or death from any cause.

## RESULTS

**Figure 1:**
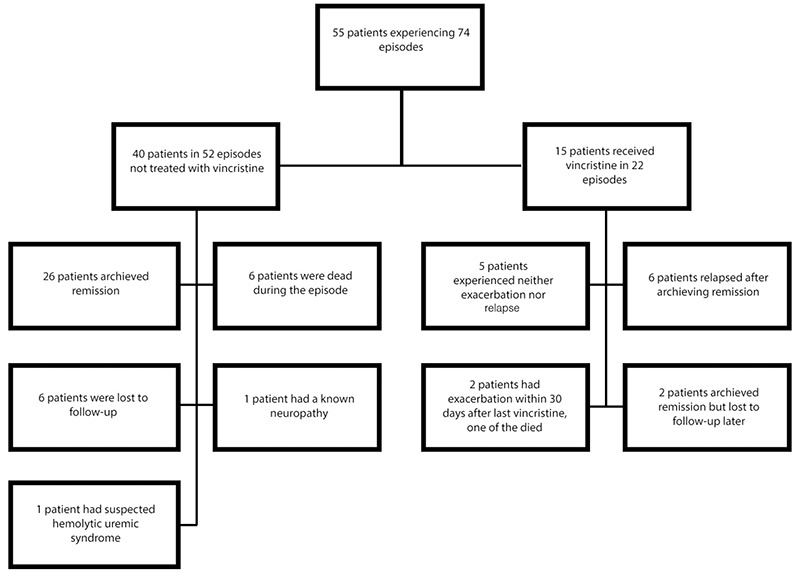
Flowchart summarizing the patients in the study.

Between October 2000 and December 2016, 55 patients presented with 74 episodes of TTP (Figure 1). Forty patients were not treated with vincristine during the given time period. Twenty-six patients experienced their first episode and achieved remission. Six patients died during the first episode and 6 were lost to follow-up. One patient could not receive vincristine because of existing neuropathy, and one patient was later evaluated as having hemolytic uremic syndrome. Only the patient with neuropathy received rituximab as an adjunctive treatment during relapse. Corticosteroids were given to 14 of 40 patients. Vincristine was administered in 22 episodes of relapsed/refractory TTP, to 15 patients (7 women and 8 men) with a median age of 37 years (range: 26-65) at the time of the first vincristine administration. The main characteristics of the patients and episodes (demographics and presenting clinical features) are listed in Table 1. All patients had acquired TTP either at diagnosis or at relapse. The median platelet count was 13.500/mm^3^ (range: 7000-134.000), the median hemoglobin value was 8.75 g/dL (range: 6-14.3), and median serum LDH at baseline was 1155 IU/L (range: 370-2283), normal values being <250 IU/L. Two patients had high creatinine levels >1.5 mg/dL, and in 79% of episodes the patients had an abnormal urinalysis with microhematuria, hemoglobinuria, proteinuria, or casts. No patient required renal replacement therapy. Eleven patients were tested for human immunodeficiency virus and all were negative. The presenting symptoms included fever in 19%, neurological symptoms in 91%, and renal abnormalities in 80% of episodes. Two thirds of the patients required a central venous line to be successfully treated with TPE. The remaining one third completed the entire course of TPE using 2 large-bore antecubital peripheral lines. Ten patients received vincristine for the initial episode of TTP refractory to TPE. Vincristine was given to 7 patients for relapsed TTP (2 for a single relapse, 5 for 2 relapses). Two patients were treated for both initial and relapsed TTP. The median time from diagnosis to the first vincristine was 10.5 days (range: 0-105). In 6 episodes, vincristine was given within 3 days after the first TPE, and these were all relapses. In 13 episodes, patients received vincristine beyond 3 days after the first TPE. In 3 episodes, the exact dates of vincristine administration were not available. The median vincristine dose per episode was 4 mg (range: 1-7 mg). Six patients in 7 episodes had an increasing platelet count within 10 days of vincristine administration. Sixty-six percent of them had been treated with vincristine within 3 days after the first TPE, whereas 2 patients in 3 episodes received vincristine beyond 3 days after the first TPE. Nine patients (60%) had normal platelet counts, discontinuation of TPE, and improvement of the TTP-associated clinical condition at week 4 after the initial infusion of vincristine. Two of the 15 patients did not achieve remission. One of the patients needed TPE within 30 days after the response and was evaluated as not achieving remission. The second patient died the following day. Remissions were achieved in 13/15 (87%) patients. Five of 15 patients (30%) experienced neither exacerbation nor relapse. The median duration of remission was 29.5 months (range: 3-105). After achieving remission, 6 patients experienced a total of 10 relapses (2 relapses in 4 patients) (8 relapse episodes) and one relapse in each of the remaining 2 patients (2 relapse episodes). The remaining 2 patients were lost to follow-up. The median time to relapse was 21 months (range: 3-99). Five of the 6 patients were retreated with vincristine and all responded to treatment. After a median follow-up from the first dose of vincristine of 55 months (range: 0.03-126), 2 patients were lost to follow-up, 11 patients were alive, and 2 patients died. Overall, in 20 of 22 episodes, a response to vincristine was achieved.

## DISCUSSION

Relapsing and/or refractory TTP is a clinical challenge. Vincristine is one of the several adjuncts to TPE. Data on vincristine use in relapsed/refractory TTP are limited to a few non-randomized studies including mostly case reports and small series (14,20). Some evidence regarding vincristine use comes from experiences with Jehovah’s Witnesses, who refuse to be treated with allogeneic blood products due to religious concerns (21,22,23). Vincristine is used in the front-line setting without TPE in this particular group of patients. We report here a cohort of patients with relapsed/refractory TTP from a single institution treated with vincristine. Although recommended after rituximab in the setting of refractory TTP, the better affordability and accessibility of vincristine and our greater experience with the drug were the reasons why we treated this rituximab-naive patient population with vincristine. Additionally, rituximab has been used off-label with increasing frequency since 2002. Our study included patients admitted before the use of rituximab. Vincristine is typically reserved for salvage therapy in patients with relapsed and refractory TTP. Patients treated with a combination of vincristine and TPE in the front-line setting were analyzed by Goldfinger and associates (24,25). They reported an improved survival rate when vincristine and TPE in combination were administered at presentation in patients treated between 1979 and 1994 (24) and then analyzed the results of a subsequent retrospective study of additional patients treated with this standardized therapeutic approach between 1995 and 2002 (25). We cannot comment on vincristine administration at presentation in patients with an initial episode, because all patients receiving early vincristine were relapsed patients in the present study. Various dosing schedules are applied for vincristine in the treatment of TTP, both in relapsed/refractory patients and in the front-line setting. Standardized regimens include TPE, corticosteroids and vincristine at a dose of 1.4 mg/m^2^ on days 1, 4, 7, and 10 in the refractory setting (4) or 1.5 mg/week for a total of 4 times (14) or vincristine 1-2 mg up to 2 mg total dose within 24 hours 3 days after the first TPE, followed by 1 mg administered after 1 week as suggested by Mazzei et al. (24). Ziman et al. (25) treated patients at presentation with vincristine 1.4 mg/m^2^ (up to 2 mg total dose) after the first TPE. Vincristine was given at 2 mg on the first day followed by 1 mg on days 4 and 7 by Ferrara et al. (15) and after 1-week off, a second course was repeated. Bobbio-Pallavicini et al. (18) treated patients who were refractory to TPE with vincristine 2 mg weekly. Vincristine was also used in combination with cyclophosphamide in addition to TPE in relapsed patients (26). The dosing frequency of vincristine plays a central role in the development of neuropathy, and giving the drug twice or thrice weekly may be more toxic than the 3-week schedule generally administered in lymphomas. The patients in the present study were treated with vincristine intravenously on days 1, 4, and 7 following TPE. A second course was given after a 1-week break in 2 patients. Eleven patients were treated concomitantly with corticosteroids in 15 episodes. Grade 1 and 2 sensory neuropathy was the predominant side effect. Vincristine was generally well tolerated without significant complications during acute and follow-up periods. Ziman et al. (25) performed a literature review of TTP patients treated with vincristine and divided them into 2 groups. Patients who received vincristine within 3 days of diagnosis (early vincristine receivers) were compared with those who were given vincristine beyond 3 days after initiation of TPE. Their statistical comparisons also included patients not treated with vincristine. Durable remission rates were significantly better among early vincristine receivers when compared with late receivers and with those who were not treated with vincristine (p=0.01 and p=0.05). In the present study, 5 patients received vincristine for 6 episodes within 3 days of TPE, and these were all relapsed patients. The median duration of remission of these episodes was 22.5 months. Importantly, vincristine appears to be at least as effective in patients previously treated for an acute episode as in those who are vincristine-naive. All previously treated patients in the present study achieved remission after retreatment with vincristine. Ziman et al. (25) reported that all the patients who relapsed after initial vincristine and TPE and were retreated with the same combination and with or without adjunctive therapies (steroids and splenectomy) achieved durable remissions. Sennett and Conrad (8) also reported a relapsed patient retreated successfully with vincristine. In the study conducted by Ferrara et al. (15) all patients retreated with vincristine achieved remission. These data are consistent with our findings. Retreatment of patients with vincristine at relapse might be considered. Therapy with corticosteroids and other immunosuppressive agents in addition to TPE may benefit a subgroup of patients with severe acquired ADAMTS13 deficiency (27). ADAMTS13 activity was measured in 6 of our patients at the time of the initial episode or during remission, and 5 were found to have increased inhibitor titers. Three of these 5 patients with increased inhibitor levels experienced neither exacerbation nor relapse. Patients with severe ADAMTS13 deficiency and high inhibitor titers may benefit more from vincristine treatment. In conclusion, vincristine provided durable responses in some patients. Further studies evaluating vincristine in the front-line setting and in the relapsed/refractory setting are needed to validate the role of vincristine in TTP patients. Based on previous studies and our findings, vincristine offers a reasonable option for treatment of patients with relapsed/refractory TTP.

## Figures and Tables

**Table 1:**
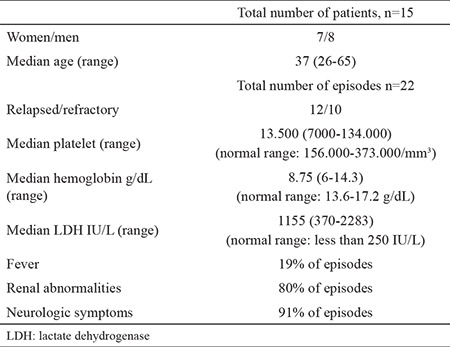
Demographic and clinical characteristics of 15 patients treated with vincristine in 22 episodes
